# Personal Protective Equipment Use by Dairy Farmworkers Exposed to Cows Infected with Highly Pathogenic Avian Influenza A(H5N1) Viruses — Colorado, 2024

**DOI:** 10.15585/mmwr.mm7344a2

**Published:** 2024-11-07

**Authors:** Kristen E. Marshall, Cara C. Drehoff, Nisha Alden, Sophia Montoya, Ginger Stringer, Allison Kohnen, Alexandra Mellis, Sascha Ellington, Jordan Singleton, Carrie Reed, Rachel Herlihy, Colorado Field Team, Colorado Field Team, Rachel Alade, Marlee Barton, Cindy Camarillo, Lauren Duval, Rebecca Hermann, Frankie Lupercio, Leovi Madera, Pamela Pagano, Jeannette Rodriguez

**Affiliations:** ^1^Colorado Department of Public Health and Environment; ^2^Career Epidemiology Field Officer Program, CDC; ^3^Epidemic Intelligence Service, CDC; ^4^Influenza Division, National Center for Immunization and Respiratory Diseases, CDC.; Epidemic Intelligence Service, CDC; Colorado Department of Public Health and Environment; Colorado Department of Public Health and Environment; Colorado Department of Public Health and Environment; Colorado Department of Public Health and Environment; Colorado Department of Public Health and Environment; Colorado Department of Public Health and Environment; Influenza Division, National Center for Immunization and Respiratory Diseases, CDC; Colorado Department of Public Health and Environment

**Summary**

**What is already known about this topic?**

Use of personal protective equipment (PPE) by farmworkers can protect them when they are working with highly pathogenic avian influenza A(H5N1)–infected cows.

**What is added by this report?**

Dairy farmworkers in Colorado who were interviewed about PPE use during work activities with ill cows reported 28% higher use of PPE after detection of A(H5N1) virus on the farm than before detection, including a 40% increase in reported use of eye protection during milking. Reported use of respirators and other masks was low.

**What are the implications for public health practice?**

Establishing strong relationships between public health agencies and agricultural organizations to communicate public health risk and protective practices on U.S. farms after detection of A(H5N1) in cows, and early distribution of PPE before A(H5N1) virus detection, might increase PPE use once an A(H5N1) outbreak is identified.

## Abstract

The risk for transmission of highly pathogenic avian influenza A(H5N1) virus from dairy cows to humans is currently low; however, personal protective equipment (PPE) use during work activities on dairy farms has not been well described. PPE use can protect farmworkers when they are working with highly pathogenic avian influenza A(H5N1)–infected cows. The Colorado Department of Public Health and Environment (CDPHE) and the Colorado Department of Agriculture (CDA) offered PPE to all Colorado farms before or during an A(H5N1) outbreak in cows in 2024. CDPHE surveyed 83 dairy workers from three farms with a confirmed bovine A(H5N1) outbreak. Frequently reported farm worker activities included milking cows or working in the milking parlor (51%), cleaning cow manure (49%), and transporting cows (46%). Frequently reported PPE items available to workers before A(H5N1) outbreaks included gloves (88%), eye protection (e.g., safety glasses or goggles) (76%), rubber boots or boot covers (71%), and head covers (69%). N95 respirator use was low among workers who were exposed to ill cows after detection of A(H5N1) virus (26%). PPE use while working with ill cows increased a mean of 28% after detection of A(H5N1) virus on surveyed farms; use of eye protection while milking cows increased the most (40%). Public health PPE distribution, education, and collaboration with CDA might have increased PPE use on dairy farms with A(H5N1) virus–infected cows and mitigated risk for farmworkers acquiring A(H5N1) virus.

## Investigation and Results

### Background

On April 25, 2024, Colorado detected highly pathogenic avian influenza (HPAI) A(H5N1) virus in cows on a dairy farm. During April–August 2024, an additional 63 dairy farms with A(H5N1) virus–positive cows were identified* in Colorado. Although transmission risk for A(H5N1) viruses from animals to humans is low, transmission has occurred in the United States, including to a dairy worker in Colorado in 2024 and to poultry workers in 2022 and 2024 (*1*–*5*). Whereas A(H5N1) virus–infected poultry typically experience rapid and high mortality, cows tend to recover (<2% herd mortality) and require ongoing care such as milking during illness (*6*). Exposure to ill^†^ cows and raw milk from acutely infected dairy cows poses a transmission risk to workers. CDC recommends use of personal protective equipment (PPE) by persons who are in contact with or near dairy cows, raw milk, or items that might be contaminated with A(H5N1) viruses (*5*,*7*,*8*). Recommended PPE includes fluid-resistant coveralls, an optional waterproof apron, a National Institute for Occupational Safety and Health (NIOSH)–approved particulate respirator (e.g., an N95^§^ filtering facepiece respirator [FFR]), goggles, a head or hair cover, gloves, boots or boot covers, and an optional face shield.

The Colorado Department of Public Health and Environment (CDPHE) collaborated with the Colorado Department of Agriculture (CDA) to respond to dairy farm A(H5N1) outbreaks^¶^ in Colorado. CDPHE and CDA conducted outreach to farm owners, offering site visits to educate and advise them about protecting dairy farmworkers from ill cows. A letter with information on A(H5N1) prevention, PPE best practices, and a PPE order link was sent to dairy facilities and industry partners (*7*). The link was publicly available on CDA’s website for ordering a free 1-month supply of N95 FFRs, surgical masks, face shields, goggles, and nitrile gloves to all Colorado farms before or during an outbreak.

Dairy farmworkers complete numerous tasks involving close contact with cows, but their specific duties and PPE use are not well described. This report includes survey results describing work activities and PPE use on three dairy farms in Colorado before and after A(H5N1) outbreaks were identified.

### Farm Participation

Colorado dairy farms with A(H5N1) virus–infected cows during July–August 2024 were eligible to participate in the retrospective survey. CDPHE contacted management at 43 affected dairy farms by telephone. Farm management gauged worker interest and scheduled CDPHE interview site visits. Individual workers voluntarily participated in interviews during site visits. Three farms located <50 miles apart in the same region of Colorado were included as a convenience sample (approximately 250 total workers). One farm included four smaller farms, which were considered a single farm for the project. One participating farm had preordered PPE before A(H5N1) virus detection, and the other two farms received PPE during their initial public health site visit, after A(H5N1) virus detection. All three farms opted to receive public health site visits upon detection. Thirty-seven (64%) of the 58 affected farms also ordered PPE. This activity was reviewed by CDC, deemed not research, and was conducted consistent with applicable federal law and CDC policy.**

### Data Collection and Analysis Methods

CDPHE conducted voluntary worker surveys in English or Spanish during site visits to the three participating farms using a structured interview questionnaire. Workers participated anonymously and received a gift card incentive for completing the survey. Interviews gathered information on work exposure, including contact with ill cows, work duties, and PPE use, both in the weeks before and after A(H5N1) virus detection on the farm. No worker or interview information was shared with employers. Summary statistics described work activities and PPE use, and adjacency matrix heat mapping methods compared the two variable group frequencies to identify clusters and overlapping trends. All analyses were conducted using R statistical software (version 2024.09.0; R Foundation).

### Participating Farm Workers

During July–August 2024, CDPHE interviewed 83 (34% of approximately 250 total employees) dairy farmworkers; among these, 72 (87%) were interviewed in Spanish. Interviews were conducted a median of 48 days after infected cows were reported to public health (IQR = 47–49 days). Among interviewed dairy workers, 44 (53%) reported exposure to ill cows starting the week before A(H5N1) virus was first detected in the cows.

### Reported Work Duties

The most commonly reported work duties were milking cows or working in the milking parlor (51%), cleaning cow manure (49%), and transporting cows (46%) (Table 1). The least commonly reported duties included repair or maintenance and hospital pen work (20%), breeding and artificial insemination (18%), and milk transport (12%).

**TABLE 1:** Work duties reported on dairy farms with highly pathogenic avian influenza A(H5N1) virus infections in cows — Colorado, 2024

Work duties performed	No. of workers (%)*		
	Total surveyed N = 83	Did not report work with ill^†^ cows n = 39	Reported work with ill cows the week before A(H5N1) identification n = 44
Milk cows or work in milking parlor	**42 (51)**	25 (60)	17 (40)
Clean cow manure or feces	**41 (49)**	26 (63)	15 (37)
Transport cows	**38 (46)**	26 (63)	12 (32)
Feed or water cows	**33 (40)**	21 (64)	12 (36)
Clean milk parlor or tanks	**33 (40)**	22 (67)	11 (33)
Vaccinate or medicate cows	**31 (37)**	23 (74)	8 (26)
Work in the calf pens	**29 (35)**	16 (55)	13 (45)
Work in the maternity pens	**25 (30)**	13 (52)	12 (48)
Conduct milk tank checks	**25 (30)**	18 (72)	7 (28)
Clean or replace cow bedding	**21 (25)**	16 (76)	5 (24)
Calving	**18 (22)**	10 (56)	8 (44)
Other duties^§^	**17 (20)**	5 (29)	12 (71)
Breeding or artificial insemination	**15 (18)**	13 (87)	2 (13)
Transport milk	**10 (12)**	5 (50)	5 (50)
No. of duties performed, median (IQR)	**4 (2–6)**	5 (3–7)	3 (2–4)

* Percentages calculated from among the total number of workers reporting the work task.

^†^ Once A(H5N1) was detected on a farm, any ill-appearing cow was considered potentially infected with A(H5N1).

^§^ Other duties included repair or maintenance, working in hospital pens, tractor operation, machine operation, and hoof trimming.

Persons working in a milking parlor might be responsible for disinfecting and drying teats and attaching and removing milking equipment. Farms might have one parlor in which all cows are milked or have a separate parlor for only ill animals for isolation and biosecurity. Manure management can involve being near cattle and contact with urine and feces. Transporting cows includes moving cows around the farm, loading cows onto vehicles, and operating vehicles. Although moving cows is mainly carried out from a distance (cows typically retreat from a worker approach), this activity occasionally does involve direct cow contact. The survey also identified a decrease in the median number of work duties performed once A(H5N1) virus was detected (from five to three activities).

### Reported PPE Use

When asked about their access to individual elements of PPE before outbreak detection, 88% of workers reported access to gloves, 76% reported access to eye protection such as safety glasses or goggles, 71% reported access to rubber boots or boot covers, and 69% reported access to head covers. Reported use of many individual PPE items was higher among dairy workers who reported exposure to ill cows in the week before or week after the detection of A(H5N1) on the farm, compared with those who did not report exposure to ill cows (Table 2). Dairy workers exposed to ill cows during the week after A(H5N1) virus detection reported higher use of gloves (93%), boots or boot covers (83%), head or hair covers (79%), and eye protection (76%) compared with those who reported exposure to ill cows in the week before detection of HPAI A(H5N1). Reported use of N95 FFRs or other respirators and other types of masks was low (9% and 27%, respectively) among workers exposed to ill cows the week before A(H5N1) outbreaks were detected, with higher usage reported among exposed workers in the week after outbreak detection (26% and 36%, respectively). Use of all CDC-recommended PPE was low among workers both in the weeks before (2%) and after (5%) A(H5N1) virus detection. Workers also reported use of items such as sunglasses and bandanas or gaiters; these items are not recommended PPE.

**TABLE 2:** Personal protective equipment and other items used by dairy farmworkers exposed to ill* cows on dairy farms with highly pathogenic avian influenza A(H5N1) virus infections in cows (N = 83)^†^ — Colorado, 2024

PPE and other items worn	No. of workers (%)		
	No exposure to ill cows n = 39	Exposure to ill cows during the week before detection of A(H5N1)^†,§ ^n = 44	Exposure to ill cows during the week after detection of A(H5N1)^†,§ ^n = 42
Gloves	32 (82)	40 (91)	39 (93)
Rubber boots or boot covers	23 (59)	35 (80)	35 (83)
Head or hair cover	23 (59)	33 (75)	33 (79)
Eye protection^¶^	27 (69)	28 (64)	32 (76)
Waterproof apron	6 (15)	18 (41)	15 (36)
Bandana or gaiter**	16 (41)	18 (41)	18 (43)
Sunglasses^¶,^**	20 (51)	14 (32)	12 (29)
Coveralls	8 (21)	13 (30)	17 (40)
Other type of (unspecified) face mask	16 (41)	12 (27)	15 (36)
N95 FFR or other respirator	6 (15)	4 (9)	11 (26)
Other type of PPE^††^	2 (5)	1 (2)	5 (12)
Used all recommended PPE^§§^	—	1 (2)	2 (5)
No PPE use reported	1 (3)	2 (5)	1 (2)
No. of PPE items used, median (IQR)	5 (3–6)	5 (4–6)	6 (5–7)

**Abbreviations:** FFR = filtering facepiece respirator; NIOSH = National Institute for Occupational Safety and Health; PPE = personal protective equipment.

* Once A(H5N1) was detected on a farm, any ill-appearing cow was considered potentially infected with A(H5N1).

^†^ The 83 interviewed workers included the 39 who had no exposure to ill cows plus the 44 who had exposure the week before detection of A(H5N1) on the farm (the first two columns of workers). The 42 workers who had exposure to ill cows the week after detection of A(H5N1) (the third column) includes all those with exposure before A(H5N1) detection minus two workers who no longer worked with ill cows after detection of A(H5N1) on the farm.

^§^ Work with ill cows began during the week before A(H5N1) was detected on the farm.

^¶^ Eye protection in the survey included safety glasses or goggles.

** These items are not CDC- or NIOSH-approved PPE items but were reported by surveyed workers.

^††^ Other reported items included long sleeves (two) and face shields (three).

^§§^ CDC-recommended PPE items include fluid-resistant coveralls, an optional waterproof apron, NIOSH-approved particulate respirator (e.g., an N95 FFR), eye protection, a head or hair cover, gloves, boots or boot covers, and an optional face shield.

Adjacency matrix frequencies identified a group of work duties with the highest reported PPE use in the weeks before and after A(H5N1) virus detection (Figure). Workers transporting cows, cleaning cow manure, and milking cows reported the highest frequency of use of gloves, rubber boots or boot covers, head covers, and eye protection. The mean use frequency of these PPE items while performing the same activities increased 28% during the week following detection of A(H5N1) virus. The largest increase in reported use frequency of eye protection from the week before to the week after detection of A(H5N1) occurred in workers who milk cows (40%). The highest correlations of work duty and PPE use (24 workers) were wearing gloves while transporting cows and wearing gloves while cleaning manure or feces (Figure).

**FIGURE:**
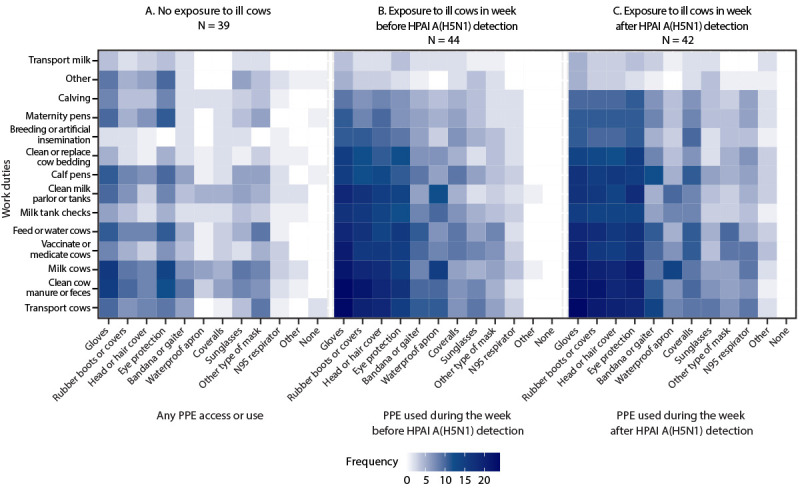
Dairy farm work duties and personal protective equipment and other items used by workers on farms with no exposure to ill* cows (A), with exposure to ill cows in the week before detection of highly pathogenic avian influenza A(H5N1) virus (B), and with exposure to ill cows in the week after A(H5N1) virus detection (C) — Colorado, 2024 **Abbreviations**: HPAI = highly pathogenic avian influenza; PPE = personal protective equipment. * Once HPAI A(H5N1) was detected on a farm, any ill-appearing cow was considered potentially infected with HPAI A(H5N1).

## Discussion

The lower mortality rate of cows infected with highly pathogenic avian influenza A(H5N1) viruses compared with that of birds can result in prolonged dairy farm worker exposures to ill cows through poorly understood transmission routes. Reported PPE use among workers at three dairy farms with A(H5N1) influenza outbreaks was high for some items, with the largest increase reported in frequency of use of eye protection once outbreaks were detected. Milking cows, the most frequently reported work duty by interviewed workers, is thought to pose higher risk for cow-to-human A(H5N1) virus transmission because of exposure to raw milk (*6*). A previous human case of A(H5N1) in a dairy worker identified in Michigan reported milk splashing into the eyes before receiving a positive A(H5N1) test result (*9*). The reported increase in eye protection among workers milking cows after A(H5N1) detection is important for protecting against A(H5N1) exposure. Dairy farms might isolate ill cows to reduce transmission, but the occurrence of asymptomatic A(H5N1) virus infections in cows means that workers can still be exposed during the milking process, highlighting the need for and importance of PPE during milking for all cows on dairy farms with A(H5N1) virus infections detected (*10*).

Reported use of N95 FFRs and other types of masks was low during most work activities. Dairy farmworkers’ duties often involve exposure to manure or milk that can contaminate respirators and masks, which might result in lower worker compliance with use as well as possible influenza A(H5N1) exposure despite wearing a mask. Hot weather and humid environments found in milking parlors can also make wearing respirators and masks uncomfortable, potentially reducing the likelihood of their use by workers carrying out farm activities, especially during hot summer months.

In this analysis, other types of masks were used more frequently than were N95 FFRs. Development of messaging by public health agencies that is consistent with CDC PPE recommendations (*7*) would help to educate farm owners and workers about the risks associated with caring for ill dairy cows on farms with A(H5N1) detected and could encourage recommended respirator use. Additional data are needed to guide recommendations for PPE use to protect worker health in these environments. Engaging dairy industry representatives and producers to recommend practices limiting worker contact with dairy cattle and their milk, along with PPE use recommendations in high-risk scenarios such as milking, might increase PPE use.

### Limitations

The findings in this report are subject to at least two limitations. First, some PPE use before A(H5N1) outbreaks might have been in response to work conditions (e.g., wearing gloves to protect hands during work with rough materials or wearing hair or head covering to protect from sun exposure) and unrelated to A(H5N1) exposure. Second, high reported PPE use might be correlated with farm engagement with public health. Dairy farms that cooperate with public health might be more likely to request PPE for workers and participate in public health investigations. All three participating farms received PPE from public health before or immediately after the detection of A(H5N1) virus on their farms.

### Implications for Public Health Practice

Public health agencies should continue to conduct outreach to farms and educate farm owners about the importance of workers using PPE during farm duties and exposure to ill cows, as well as understanding and reporting signs of human illness during A(H5N1) herd outbreaks. Collaboration with state agricultural partners can strengthen relationships and public health practice at dairy farms. As the A(H5N1) outbreak in dairy herds evolves, providing PPE to farms before outbreaks occur might help increase PPE use, especially during high-risk activities such as milking, and prevent human cases of A(H5N1).

## Footnotes

*
https://ag.colorado.gov/animal-health/reportable-diseases/avian-influenza/hpai-in-dairy-cattle

† Once A(H5N1) was detected on a farm, any ill-appearing cow was considered potentially infected with A(H5N1).

§ N95 and NIOSH-approved are certification marks of the U.S. Department of Health and Human Services registered in the United States and several international jurisdictions.

¶ A(H5N1) outbreaks are detected by farms reporting ill cows with laboratory confirmation of A(H5N1) infection, or through bulk tank milk testing. All farms with an A(H5N1) outbreak had more than one ill cow.

** 45 C.F.R. part 46.102(l)(2), 21 C.F.R. part 56; 42 U.S.C. Sect. 241(d); 5 U.S.C. Sect. 552a; 44 U.S.C. Sect. 3501 et seq.

## References

[1] Drehoff CC, White EB, Frutos AM, ; H5N1 Field Investigation Team. Cluster of influenza A(H5) cases associated with poultry exposure at two facilities—Colorado, July 2024. MMWR Morb Mortal Wkly Rep 2024;73:734–9. 10.15585/mmwr.mm7334a1 39207932 PMC11361414

[2] Uyeki TM, Milton S, Abdul Hamid C, Highly pathogenic avian influenza A(H5N1) virus infection in a dairy farm worker. N Engl J Med 2024;390:2028–9. 10.1056/NEJMc2405371 38700506

[3] CDC. CDC newsroom: CDC reports fourth human case of H5 bird flu tied to dairy cow outbreak [Press release]. Atlanta, GA: US Department of Health and Human Services, CDC; 2024. https://www.cdc.gov/media/releases/2024/p-0703-4th-human-case-h5.html

[4] CDC. CDC newsroom: CDC reports second human case of H5 bird flu tied to dairy cow outbreak [Press release]. Atlanta, GA: US Department of Health and Human Services, CDC; 2024. https://www.cdc.gov/media/releases/2024/s0522-human-case-h5.html

[5] Garg S, Reed C, Davis CT, Outbreak of highly pathogenic avian influenza A(H5N1) viruses in U.S. dairy cattle and detection of two human cases—United States, 2024. MMWR Morb Mortal Wkly Rep 2024;73:501–5. 10.15585/mmwr.mm7321e1 38814843 PMC11152367

[6] American Veterinary Medical Association. Avian influenza virus type A (H5N1) in U.S. dairy cattle. Schaumburg, IL: American Veterinary Medical Association; 2024. https://www.avma.org/resources-tools/animal-health-and-welfare/animal-health/avian-influenza/avian-influenza-virus-type-h5n1-us-dairy-cattle

[7] CDC. Avian influenza (bird flu): interim guidance for employers to reduce the risk of novel influenza A for people working with or exposed to animals. Atlanta, GA: US Department of Health and Human Services, CDC; 2024. https://www.cdc.gov/bird-flu/prevention/worker-protection-ppe.html

[8] US Department of Agriculture, Animal and Plant Health Inspection Service. Highly pathogenic avian influenza (HPAI) H5N1 personal protective equipment recommendations—May 29, 2024. Washington, DC: US Department of Agriculture; 2024. https://www.aphis.usda.gov/sites/default/files/hpai-ppe-recommendations.pdf

[9] Michigan Department of Health and Human Services. Additional influenza A (H5) case detected in Michigan [Press release]. Lansing, MI: Michigan Department of Health and Human Services; 2024. https://www.michigan.gov/mdhhs/inside-mdhhs/newsroom/2024/05/30/h5n1-updates

[10] US Department of Agriculture. USDA actions to protect livestock health from highly pathogenic H5N1 avian influenza [Press release]. Washington, DC: United States Department of Agriculture; 2024. https://www.usda.gov/media/press-releases/2024/04/24/usda-actions-protect-livestock-health-highly-pathogenic-h5n1-avian

